# A Rat Model for Muscle Regeneration in the Soft Palate

**DOI:** 10.1371/journal.pone.0059193

**Published:** 2013-03-15

**Authors:** Paola L. Carvajal Monroy, Sander Grefte, Anne M. Kuijpers-Jagtman, Maria P. A. C. Helmich, Dietmar J. O. Ulrich, Johannes W. Von den Hoff, Frank A. D. T. G. Wagener

**Affiliations:** 1 Department of Orthodontics and Craniofacial Biology, Nijmegen Centre for Molecular Life Sciences, Radboud University Nijmegen Medical Centre, Nijmegen, The Netherlands; 2 Department of Biochemistry, Nijmegen Centre for Molecular Life Sciences, Radboud University Nijmegen Medical Centre, Nijmegen, The Netherlands; 3 Department of Plastic and Reconstructive Surgery, Radboud University Nijmegen Medical Center, Nijmegen, The Netherlands; The University of Tennessee Health Science Center, United States of America

## Abstract

**Background:**

Children with a cleft in the soft palate have difficulties with speech, swallowing, and sucking. Despite successful surgical repositioning of the muscles, optimal function is often not achieved. Scar formation and defective regeneration may hamper the functional recovery of the muscles after cleft palate repair. Therefore, the aim of this study is to investigate the anatomy and histology of the soft palate in rats, and to establish an *in vivo* model for muscle regeneration after surgical injury.

**Methods:**

Fourteen adult male Sprague Dawley rats were divided into four groups. Groups 1 (n = 4) and 2 (n = 2) were used to investigate the anatomy and histology of the soft palate, respectively. Group 3 (n = 6) was used for surgical wounding of the soft palate, and group 4 (n = 2) was used as unwounded control group. The wounds (1 mm) were evaluated by (immuno)histochemistry (AZAN staining, Pax7, MyoD, MyoG, MyHC, and ASMA) after 7 days.

**Results:**

The present study shows that the anatomy and histology of the soft palate muscles of the rat is largely comparable with that in humans. All wounds showed clinical evidence of healing after 7 days. AZAN staining demonstrated extensive collagen deposition in the wound area, and initial regeneration of muscle fibers and salivary glands. Proliferating and differentiating satellite cells were identified in the wound area by antibody staining.

**Conclusions:**

This model is the first, suitable for studying muscle regeneration in the rat soft palate, and allows the development of novel adjuvant strategies to promote muscle regeneration after cleft palate surgery.

## Introduction

Cleft lip and/or palate (CLP) is the most common congenital facial malformation in humans. It occurs in about 1∶500 to 1∶1000 births, with ethnic and geographic variation [1]. CLP is generally divided into clefts involving the lip with or without cleft palate, and isolated cleft palate [2]. In 20 to 34% of the cases it is part of a syndrome, and associated with other congenital defects [3]. About 45% of all patients with CLP have a cleft of the soft palate [4].

The levator veli palatini is the major muscle of the soft palate, which moves it up and down. This muscle is therefore critical for the functioning of the soft palate during speech, swallowing, and sucking. Children with a cleft palate can not separate the nasal from the oral cavity during speech, a phenomenon known as velopharyngeal dysfunction [5], [6]. The surgical repair of the soft palate normally takes place early in childhood at 6–36 months of age, although the protocols are highly variable [2]. Surgery is required to close the defect and to reconstruct the palatal muscles [7]. The aim is to restore the function of the soft palate allowing normal speech development [8], [9]. However, velopharyngeal dysfunction persists in 7 to 30% of the patients, despite anatomical repositioning of the muscles during surgery, [5], [6], [10]–[12]. This results in speech abnormalities [13]. Various factors such as age at the time of the surgery, skills and experience of the surgeon, type and extension of the cleft, and damage of the motor and proprioceptive nerves have been attributed to suboptimal repair [5], [14]–[16].

In general, muscle tissue possesses a large ability to regenerate. Satellite cells (SatCs) are the primary muscle stem cells, and responsible for postnatal muscle growth, maintenance, and repair [17]. Upon injury, SatCs are activated and migrate to the wound, proliferate, differentiate, and form new myofibers or repair damaged ones [18]. SatCs are located between the basal lamina and the plasma membrane [17], [19], and express the transcription factor Pax7 [20], [21]. A distinct gene expression profile characterizes the SatC progeny [22], [23]. The myogenic determination factor 1 (MyoD) is expressed during SatC proliferation, whereas differentiation is marked by a decline in Pax 7 expression, and the induction of myogenin (MyoG) [24]. Differentiating myoblasts express various genes that encode structural proteins such as myosin heavy chain (MyHC), and finally fuse to form myotubes [25], [26]. SatC differentiation and, hence, muscle repair is regulated by signaling molecules from infiltrating macrophages, injured myofibers, and the disrupted extracellular matrix [18], [27].

Several strategies have been used in regenerative medicine to improve muscle regeneration. Growth factors, satellite cells, biological and synthetic scaffolds, or a combination of these have been applied to injured muscles with varying results [28]–[32]. Most studies on muscle regeneration, however, have been performed in limb, trunk, or cardiac muscles, while studies on head muscles are scarce. Skeletal muscles from the trunk and limbs are derived from the somites during embryonic development [33], while most head muscles, including those of the soft palate, are derived from the branchial arches [34]–[36]. Interestingly, head muscles generally contain less SatCs than limb muscles [37]. Head muscles also regenerate much slower than limb muscles after freeze, crush or similar injuries, and more fibrous connective tissue is generally formed during healing [38]. Proliferating SatCs from head muscles also express a different profile of transcription factors [37]. In addition, the muscles in the soft palate of CLP patients are smaller than normal palatal muscles and the myofibers are not properly organized [39], [40]. All this may contribute to the poor regeneration of soft palate muscles after surgical closure of the soft palate [41]. Taken together, scar formation and incomplete muscle regeneration seem to be the main causes of muscle dysfunction after cleft palate repair, next to the already mentioned factors.

Up to now, no animal models are available to investigate muscle regeneration in the soft palate. Therefore, the aim of this study was to describe the anatomy and histology of the soft palate in rats, and to establish an *in vivo* model for muscle regeneration in the soft palate after surgical injury.

## Materials and Methods

### Animals

#### Ethics statement

Approval of the research protocol was obtained from the local Board for Animal Experiments (Dier Experimenten Commissie) from the Radboud University Nijmegen in accordance with Dutch laws and regulations (RU-DEC 2011-125).

Fourteen adult male Sprague Dawley rats, weighing 280–300 g (Harlan BV, Horst, The Netherlands) were housed under standard laboratory conditions. The rats had been acclimatized to the animal facility for one week, before the start of the experiments. The animals were divided into four groups, group 1 (n = 4) and group 2 (n = 2) to investigate the anatomy and histology of the soft palate, respectively. Group 3 (n = 6) was used for surgical wounding of the soft palate, and group 4 (n = 2) was used as unwounded control group.

### Experimental Procedures

#### Dissection techniques

Anatomical dissection of the soft palate was performed in four animals (Group 1) using an operating microscope (Carl Zeiss AG, Oberkochen, Germany) after euthanasia with CO_2_/O_2_. In the first stage, as described elsewhere [42], a ventral incision was made extending from the mandibular symphysis to the clavicle. The subcutaneous tissues were separated until the submandibular gland was visible. By removal of the salivary gland, the digastric and sternocleidomastoid muscles were then exposed. The posterior belly of the digastric muscle was dissected to its origin, and pulled laterally to expose the tympanic bulla. This procedure was conducted at both sides, and the levator veli palatini was carefully dissected from its origin to its insertion in the soft palate. In the second stage, the mandibular ramus was cut on both sides, and the soft palate was dissected to examine the origin and course of the tensor veli palatini muscle.

In group 2, the animals were decapitated after sacrifice with the standard CO_2_/O_2_ protocol, and the heads were fixed in 4% paraformaldehyde (PFA) for 24 hours at 4°C, and then rinsed in 0.1 M phosphate-buffered saline (PBS, pH 7.4). Serial coronal sections of 5 µm were cut for histochemical staining after decalcification in 10% ethylenediaminetetraacetic acid (EDTA) and paraffin embedding.

#### Excisional wounding of the soft palate

**Figure 1:**
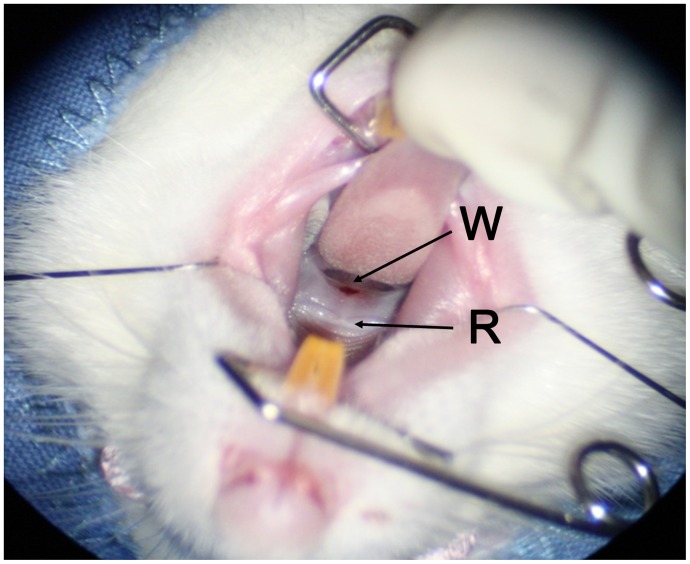
Excisional wounding of the soft palate. Excisional wounds (1 mm ø) were made in the soft palate, 7 mm behind the 9th palatal ruga. W: excisional wound, R: 9th ruga.

Six animals (group 3) received buprenorphine (0.02 mg/kg s.c.; Temgesic, Schering Plough, Brussels, Belgium) as analgesic before surgery, and also at the next two days with twelve hour intervals. General anesthesia was induced with a mixture of ketamine (75 mg/kg i.p.; Nimatek, Eurovet Animal Health B.V, Bladel, The Netherlands) and medetomidine (0.5 mg/kg i.p.; Dexdomitor, Janssen–Cilag B.V, Tilburg, The Netherlands). In addition, atropine (0.05 mg/kg i.m.; Atropine, Pharmachemie B.V, Harlem, The Netherlands) was injected to prevent medetomidine-induced bradycardia. The animals were placed in a supine position on an operating table tilted at 45° to allow optimal surgical exposure of the soft palate. Body temperature was kept at 38°C using a heating pad. All procedures were performed in semi-sterile conditions under an operating microscope (Carl Zeiss AG, Oberkochen, Germany) by the same operator (P.L.C.M) trained in microsurgical techniques. Chlorhexidine digluconate gel 0.2% (Orosol; Fresenius Kabi B.V, Schelle, Belgium) was used to clean the surgical area. Excisional wounds (1 mm ø) were made in the soft palate using a biopsy punch, seven mm behind the ninth palatal ruga (Figure 1). Reversion of the anesthesia was induced with atipamezol hydrochloride (0.5 mg/kg i.p.; Antisedan; Janssen-Cilag B.V, Tilburg, The Netherlands). The animals received powdered chow in water during the following three days. Their behavior was monitored daily with special attention for water and food intake, loss of weight, and activity. The animals were euthanized with the standard CO_2_/O_2_ protocol after seven days.

#### Histology

After euthanasia, the soft palates of the six experimental animals (group 3) and two control animals (group 4) were dissected, fixed in 4% PFA in PBS, and processed for paraffin embedding. Paraffin sections of the two heads (group 2) and the eight soft palates (group 3 and 4) were stained with azocarmine G and aniline blue (AZAN) to discriminate collagen (blue) from muscle tissue (red).

#### Immunohistochemistry

Sections were deparaffinated, rehydrated, treated with 3% H_2_O_2_ for 20 minutes to inactivate endogenous peroxidase, and post-fixed with 4% PFA in PBS. For Pax7 and MyoD staining, the sections were first heated in 0.25 mM EDTA/10 mM TRIS buffer (pH 9.0) at 100°C for 10 minutes. For MyoG staining, the sections were first heated in citrate buffer (pH 6.0) for 40 minutes at 100°C. For alpha-smooth muscle actin (ASMA) and fast myosin skeletal heavy chain (MyHC) staining, the sections were heated in citrate buffer (pH 6.0) at 70°C for 10 minutes, and subsequently treated with 0.075% trypsin in PBS (pH 7.4) for 15 minutes to retrieve antigens. Sections were then incubated with mouse anti-ASMA (1∶10.000; Chemical CO, St Louis, MO, USA), mouse anti-fast MyHC (1∶5000; Sigma Chemical CO, St Louis, MO, USA), mouse anti-Pax7 (1∶100; Developmental Studies Hybridoma Bank, Iowa City, CA USA), mouse anti-MyoD (1∶50; DAKO, Dakopatts, Glostrup, Denmark), or mouse anti-myogenin (1∶100; Developmental Studies Hybridoma Bank), overnight at 4°C. Next, the sections were incubated with biotinylated secondary antibodies donkey-anti-mouse IgG (H+L) (1∶500; Jackson Labs, West Grove, Pa, USA), and a preformed biotinylated horseradish peroxidase - avidin complex (Vector Laboratories, Burligame, CA, USA). The sections were stained with 3,3-diaminobenzidine substrate, and analyzed qualitatively.

## Results

### Anatomy of the Soft Palate Muscles

**Figure 2:**
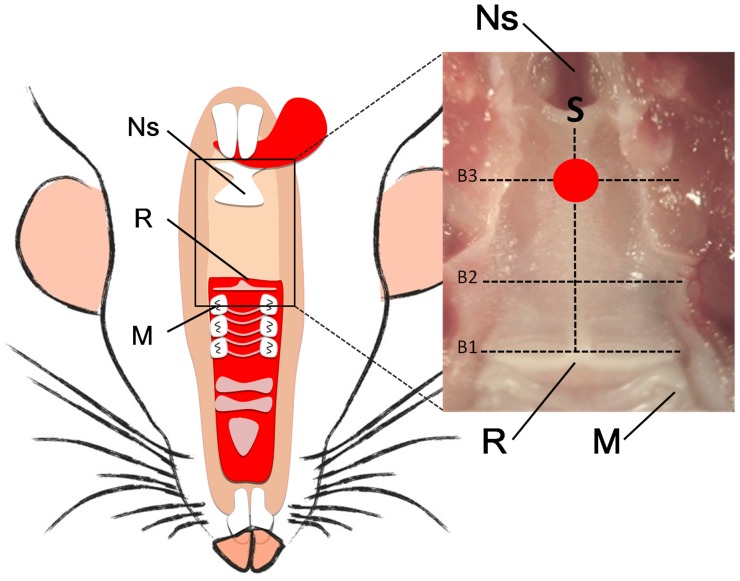
General aspect of the soft palate in rats. Left: Schematic representation of the soft palate (intraoral view). Right: The soft palate in the rat extends from the posterior edge of the hard palate (9^th^ ruga) towards the nasopharyngeal sphincter. In adult rats, the length of the soft palate is about 11 mm. Ns: nasopharygeal sphincter, R: 9th ruga, M: molar. The red circle indicates the location of the excisional wound (1 mm ø). The dotted lines indicate the location of the histological sections shown in Figure 4. S: midsagittal section. B1, B2, and B 3 coronal sections.

In adult rats, the length of the soft palate is about 11 mm, which is relatively longer than in humans. It extends from the posterior edge of the hard palate (9^th^ ruga) towards the nasopharyngeal sphincter (Figure 2).

**Figure 3:**
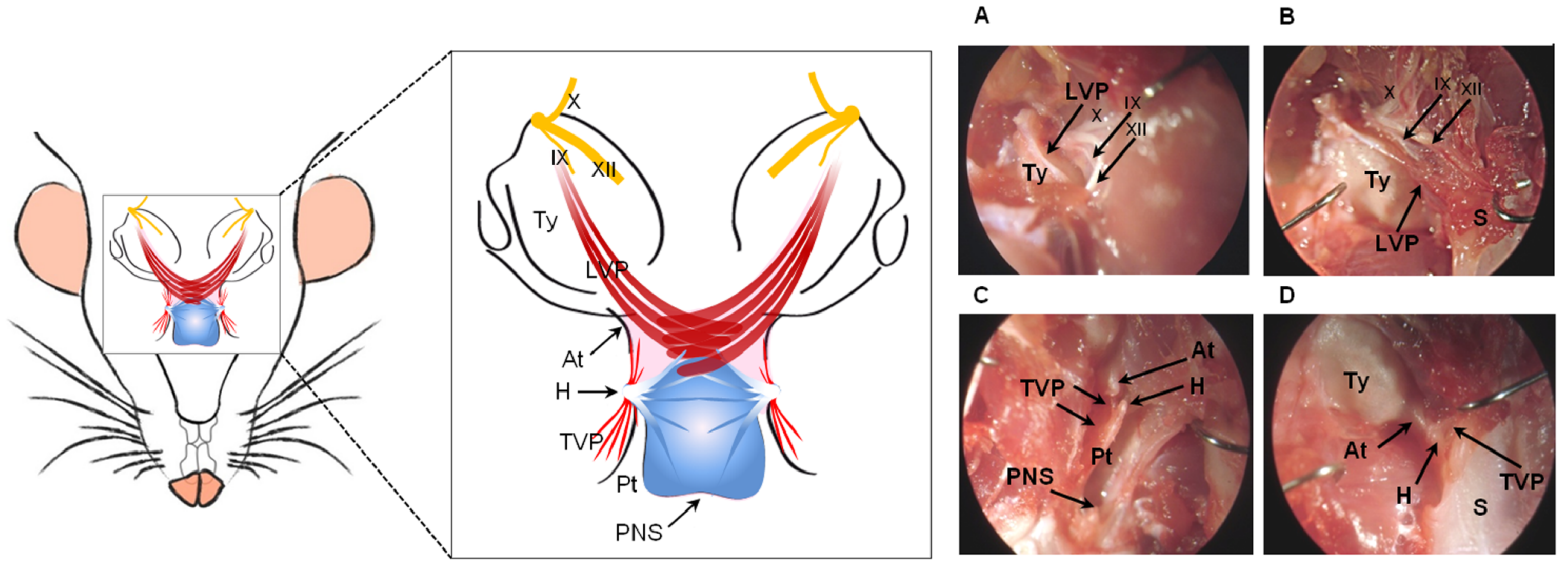
Anatomy of levator veli palatini and tensor veli palatini. Left: Schematic representation of the levator veli palatini and tensor veli palatini muscles. Right: (A) The levator veli palatini muscle arises from the inferior surface of the temporal bone, lateral and posterior from the tympanic bulla. The tympanic bulla is a bony projection of the temporal bone containing the tympanic cavity. (B) Posterior to the pterygoid process, the levator veli palatini continues towards the soft palate. The glossopharyngeal, vagus and hypoglossal nerves are visible between the levator veli palatini and the skull base. (C) The tensor veli palatini originates from the inferior surface of the sphenoid bone, the lateral surface of the perygoid plate, and the auditory tube. (D) The tendon of the tensor veli palatini turns around a curved process; the pterygoid hamulus. It continues medially towards the soft palate and forms the palatine aponeurosis. LVP: levator veli palatini muscle, TVP: tensor veli palatini muscle, IX: glossopharyngeus nerve, X: vagus nerve, XII: hypoglossus nerve, Ty: Tympanic bulla, At: auditory tube, H: pterygoid hamulus, Pt: pterygoid process, PNS: posterior nasal spine, S: soft palate.

The left part of figure 3 shows a schematic drawing of the levator veli palatini and tensor veli palatini muscles. Clinical pictures are presented in figures 3 A to D. The levator veli palatini muscle originates from the inferior surface of the temporal bone, and runs in medial and anterior direction (Figure 3A). It then runs posterior to the pterygoid process towards the soft palate, crosses the midline, and joins the contralateral levator veli palatini muscle fibers forming a muscle sling (Figure 3B). The glossopharyngeal, vagus and hypoglossal nerves are visible between the levator veli palatini and the skull base (Figure 3A and 3 B). The tensor veli palatini originates from the inferior surface of the sphenoid bone, the lateral surface of the pterygoid plate, and the auditory tube (Figure 3C). It runs towards the pterygoid process and its tendon curves around the pterygoid hamulus. It then continues medially towards the soft palate and forms the palatine aponeurosis (Figure 3D).

### Histology of the Soft Palate

**Figure 4:**
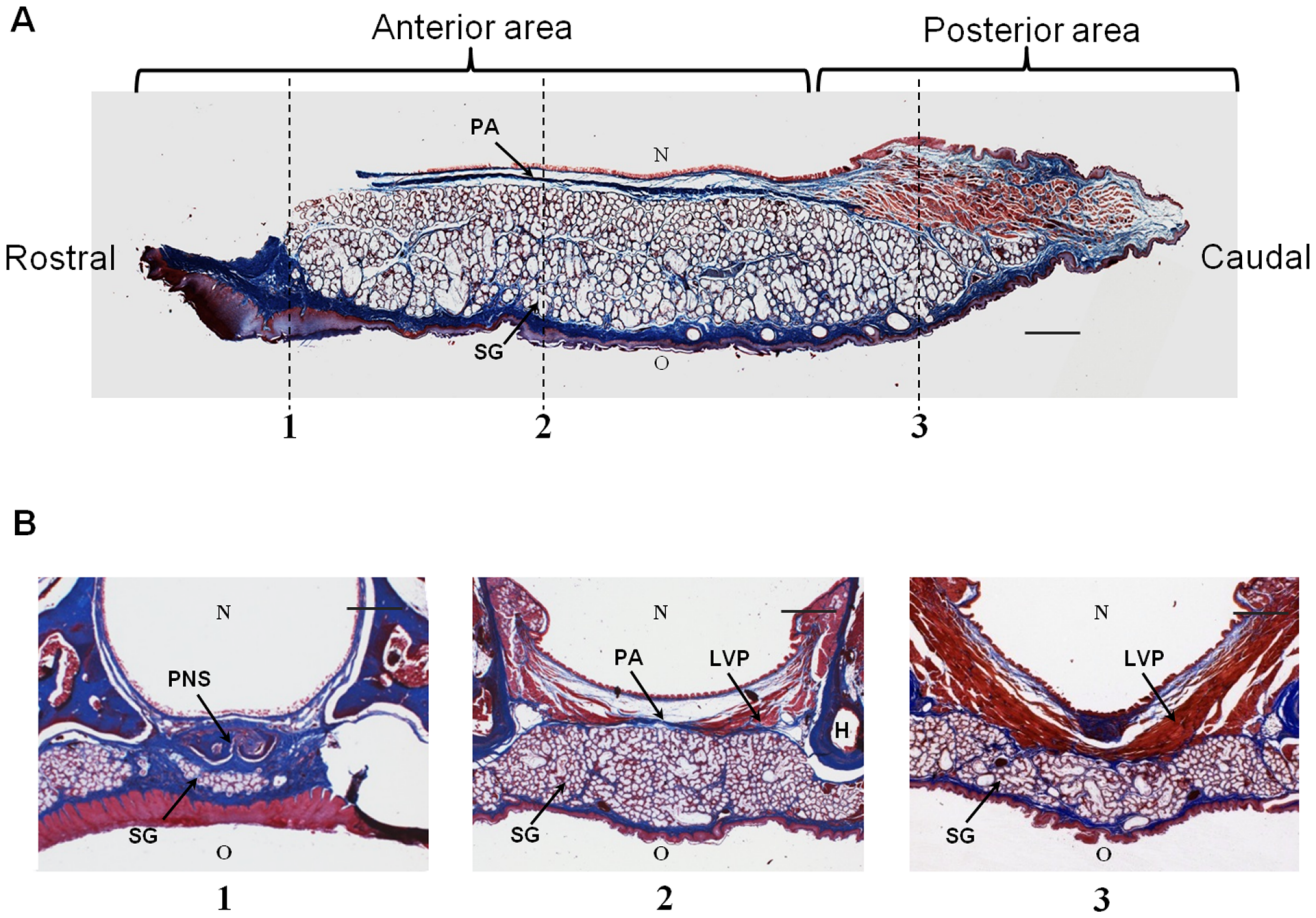
Histology of the soft palate. Paraffin sections were were cut from the tissue and stained with AZAN. (A) Midsagittal section of the soft palate. The anterior two thirds of the soft palate mainly contain salivary glands. The posterior third of the soft palate contains an additional layer of muscle tissue. (B) Coronal sections of the soft palate. The dotted lines in figure A indicate the position of the coronal sections. (B1) The palatine aponeurosis is the continuation of the tensor veli palatini and inserts into the posterior nasal spine. (B2) Posterior to the pterygoid hamulus, the levator veli platini fibers insert into the palatine aponeurosis. (B3) Most of muscle fibers of the levator veli palatini cross the midline and form a sling suspended from the skull base. N: nasal cavity, O: oral cavity, PA: palatine aponeurosis, SG: salivary glands, PNS: posterior nasal spine, LVP: levator veli palatini muscle, H: pterygoid hamulus. The bar represents 500 µm.

Two main areas were identified in the soft palate, an anterior and a posterior area (Figure 4A). The anterior area is characterized by a thick layer of salivary glands, which is covered by a layer of oral (bottom) and nasal mucosa (top) (Figure 4B-2). The oral surface is covered by a keratinized stratified squamous epithelium, while the nasal surface is covered by a pseudostratified ciliated columnar epithelium. The oral mucosa contains a thick collagenous submucosa. A thinner dense layer of collagenous tissue is present under the nasal epithelium; the palatine aponeurosis (Figure 4A). This is the continuation of the tensor veli palaini muscle, and inserts into the bony posterior nasal spine (Figure 4B-1). In the posterior area of the soft palate, the nasal epithelium changes into a stratified squamous epithelium (Figure 4A). In addition, a layer of muscle tissue is present. Posterior to the bony pterygoid hamulus, the levator veli palatini muscle fibers insert into the palatine aponeurosis (Figure 4B-2). However, most of the levator veli palatini fibers cross the midline forming a muscle sling suspended from the skull base (Figure 4B-3). The nasopharyngeal sphincter is formed by the most posterior muscle fibers in the soft palate. The sphincter mainly contains fibers from the palatoglossal and palatopharyngeal muscles. The thick glandular layer gradually becomes thinner towards the posterior part of the soft palate (Figure 4A).

### Wound Healing in the Soft Palate

**Figure 5:**
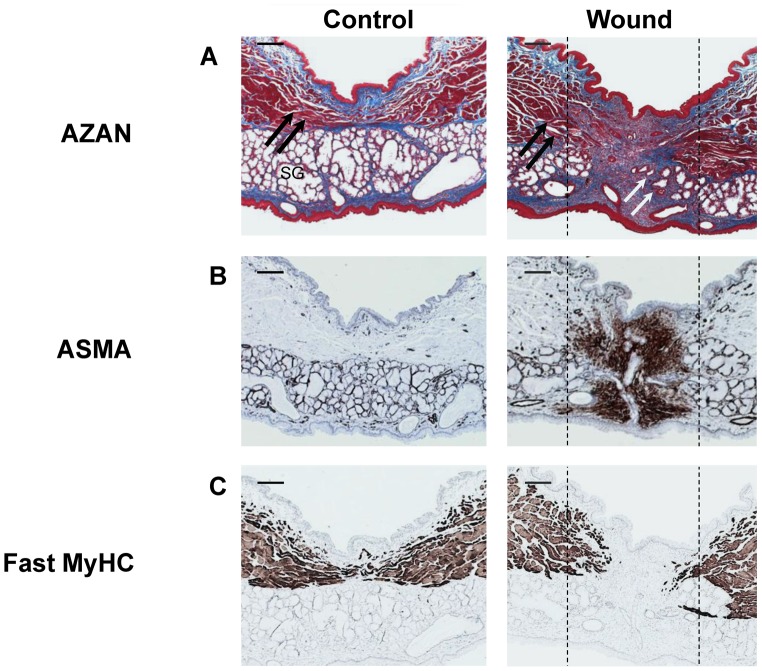
Regeneration of the soft palate after wounding. Control and wound tissues from the soft palate were stained with AZAN, and with antibodies against myofibroblasts (ASMA) and fast muscle fibers (Fast MyHC). After 7 days, extensive granulation tissue with collagen and myofibroblasts had formed. (A) AZAN staining. Connective tissue is stained blue, muscle tissue red. Black arrows indicate muscle fibers, white arrows indicate initial regeneration of salivary glands. SG: Salivary glands. (B) ASMA (Brown). Myofibroblasts were not present in the controls, except in blood vessels and salivary glands. In contrast, large numbers of myofibroblasts were present in the wound area at 7 days in the experimental group. (C) Fast MyHC (brown). In both groups, almost all myofibers were of the fast-twitch type. Wound margins are indicated by the dotted lines. The bar represents 200 µm.

Full-thickness defects (1 mm ø) were made in the soft palate of six rats (group 3), and left to heal for seven days. In addition, the soft palate of two other animals (group 4) was used as controls. All animals survived surgery, and showed no weight loss. After seven days, all wounds showed clinical healing. In the controls, both a layer of muscle tissue, and a layer of salivary glands are present (Figure 5A left). The wounded palates show loss of the major parts of these layers (Figure 5A right). Collagenous granulation tissue is present in the wound area, and only limited regeneration of salivary glands and muscles has occurred (Figure 5A).

**Figure 6:**
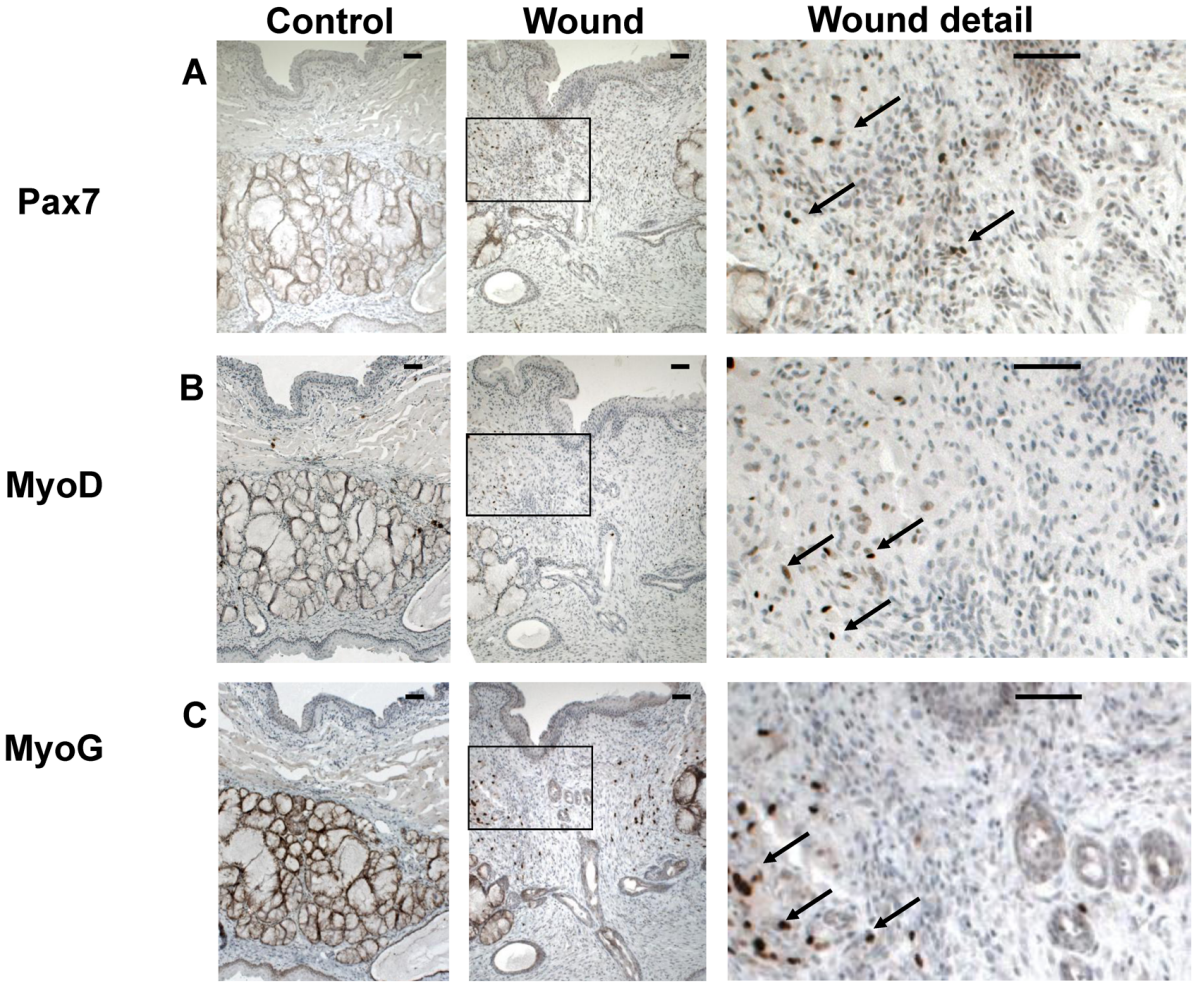
Satellite cells and myofibers after wounding. Control and wound tissues were stained with antibodies against Pax7, MyoD, and Myogenin. Large numbers of activated satellite cells and regenerating myofibers are present in the experimental group. (A) Satellite cells (arrows) express the transcription factor Pax7. (B) The myogenic determination factor 1 (MyoD, arrows) is expressed during satellite cell proliferation. (C) Differentiation is marked by a decline in Pax 7 expression, and the induction of myogenin (MyoG, arrows). Pax7-, MyoD-, and MyoG positive cells are stained brown. The bar represents 50 µm.

ASMA is a marker for myofibroblasts. Myofibroblasts were not present in the controls tissue of the controls, except in blood vessels and salivary glands (Figure 5B). In contrast, large numbers of myofibroblasts were present in the wound area at 7 days in the experimental group. In both groups, almost all myofibers were of the fast-twitch type (Figure 5C). More activated SatCs (Pax7-, MyoD-, and MyoG-positive) were present in regenerating muscle fibers in the wound edges than in control muscle (Figure 6A, B, and C).

## Discussion

Speech abnormalities are commonly observed in children with a history of a cleft in the soft palate. This can have a devastating effect on their interaction with other children and adults [15]. The regeneration of muscles in the soft palate after surgery may be hampered because of (1) their low intrinsic regenerative capacity, (2) the specific muscle properties related to clefting, and (3) the development of fibrosis [41]. The outcome of surgery may be improved by novel strategies based on tissue engineering.

A suitable animal model is required to design and optimize new strategies for soft palate repair. To our knowledge, we present the first model for the regeneration of the soft palate following surgical wounding. Our study shows that the anatomy and histology of the soft palate muscles of the rat are largely comparable to those of humans. The levator veli palatini and tensor veli palatini muscles in the rat were shown to have a similar origin and course as its homologous in humans [43]–[45]. In contrast, the muscular component of the soft palate in rats occupies only the posterior third of the tissue [44], while in humans this is about half. The levator veli palatini is the most important muscle for the elevation of the soft palate in both humans and rodents [40], [42], [44]. Together, the tensor veli palatini and the levator veli palatini also control the passage of air through the auditory tube [46], [47]. The widespread use of rodents in biomedical research ensures the availability of antibodies for specific immune staining. In combination with the ease of handling and low costs. this makes the rat the most suitable animal model.

Our model consists of a full-thickness defect in the soft palate that creates a temporal communication between the nasopharynx and the oropharynx, and thus increases the risk of bronchoaspiration. Therefore, the animals were closely monitored during the seven days after surgery. No complications were observed during this period.

Extensive collagen deposition and initial regeneration of muscle fibers and salivary glands was demonstrated after 7 days. Collagen is deposited by fibroblasts in the granulation tissue. It has been previously shown that fibroblasts proliferate in close association with satellite cells and regenerating myofibers [48]. Fibroblasts then stimulate the proliferation of satellite cells during the early phase of regeneration. Later in the regeneration process, the number of fibroblasts normally decreases to allow the formation of new muscle fibers. Growth factors such as transforming growth factor-β1 (TGF-β1) induce the differentiation of fibroblasts into myofibroblasts, which produce large amounts of extracellular matrix [49], [50]. In addition, myofibroblasts induce wound contraction. The prolonged presence of myofibroblasts in the wound leads to fibrosis [29], [49]. Since activated SatCs do not migrate into fibrotic tissue, this may impair muscle regeneration and functional recovery of the muscle tissue after injury [51].

Both fast- and slow-twitch fibers have been found in human soft palate muscles [52], [53]. In contrast, we mainly found fast-twitch fibers in the rat soft palate. However, slow-twitch fibers were also found in the more lateral parts of the levator veli palatini muscle in rats [54]. In our study, Pax7-, MyoD-, and MyoG-positive cells were found at the wound edges, demonstrating the presence of activated SatCs and differentiating myofibers. In limb muscles, activated SatCs and regenerating myofibers also appear after about one week [48], [51], [55], [56]. In limb muscles, SatCs proliferate extensively within the first 2–3 days after injury [57]. At about 5 days after injury, activated SatCs differentiate into myoblasts and fuse, which leads to restoration of the injured muscle within 10 days [57]. In contrast, injured head muscles do not restore within 12 days [38]. This indicates that the regeneration of head muscles is slower than that of limb muscles. Thus, further studies are necessary in order to fully characterize the regeneration of soft palate muscles.

### Conclusion

The presented rat model is suitable to study muscle regeneration in the soft palate after surgical injury, and allows the development of novel adjuvant strategies to promote muscle regeneration. This offers new perspectives for the treatment of CLP patients, and for various other conditions in which the regeneration of head muscles is compromised.
